# First person – Mohamadamin Forouzandehmehr

**DOI:** 10.1242/dmm.050812

**Published:** 2024-04-26

## Abstract

First Person is a series of interviews with the first authors of a selection of papers published in Disease Models & Mechanisms, helping researchers promote themselves alongside their papers. Mohamadamin Forouzandehmehr is first author on ‘ *In silico* study of the mechanisms of hypoxia and contractile dysfunction during ischemia and reperfusion of hiPSC cardiomyocytes’, published in DMM. Mohamadamin conducted the research described in this article while a doctoral candidate in the lab of Prof. Jari Hyttinen at the Computational Biophysics and Imaging Group (CBIG), Faculty of Medicine and Health Technology, Tampere University, Tampere, Finland, and is now a postdoctoral researcher in the same lab, investigating the molecular mechanisms of cardiac diseases and subcellular crosstalks in cardiomyocytes.


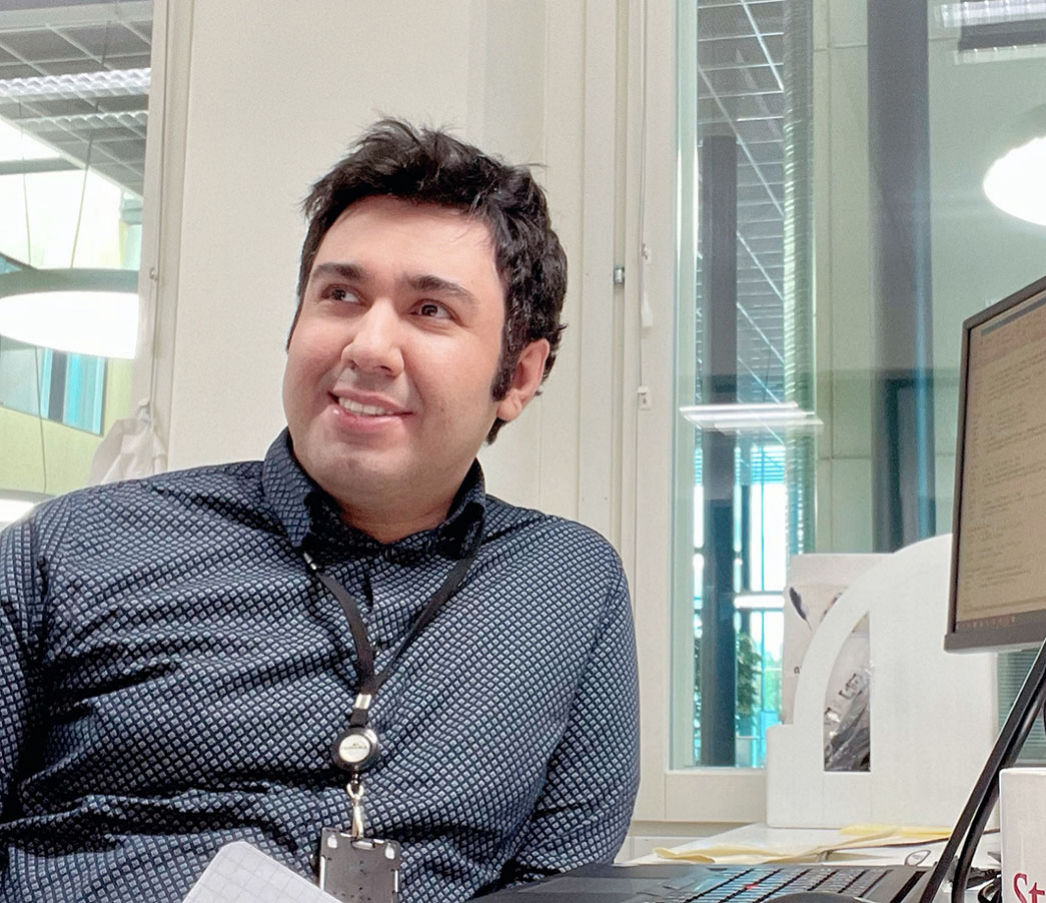


**Mohamadamin Forouzandehmehr**

**How would you explain the main findings of your paper to non-scientific family and friends?**

We investigated how a lack of oxygen (ischemia) followed by restored oxygen flow (reperfusion) affects heart muscle cells. This situation, called ischemia reperfusion (IR), can damage the heart. Scientists are increasingly interested in studying IR using special heart muscle cells grown in a lab derived from human stem cells (known as human induced pluripotent stem cell-derived cardiomyocytes, or hiPSC-CMs). We created a computer model of these heart muscle cells to understand what happens during IR. Our model considers factors like how the cells contract and relax, how they handle calcium, a crucial ion for muscle function, and how oxygen levels affect the cells, by introducing a novel oxygen dynamics formulation.

We also tested a drug called levosimendan (LEVO) in the model. LEVO has shown promise in preventing irregular heartbeats (arrhythmias) in these lab-grown heart cells when oxygen is low. The key findings of the study can be summarized as: a protein called sarcoplasmic reticulum calcium-ATPase (SERCA), important for muscle relaxation, might play a similar role in IR injury as it does in heart failure caused by infection (sepsis).

According to the model simulations, the drug LEVO seems to help muscles relax during IR by affecting how calcium interacts with muscle proteins, not by directly affecting SERCA. Furthermore, this research suggests that malfunctioning SERCA might be a key player in IR damage.

Our study provides new details on how SERCA malfunctions during IR. The model suggests that specific sites within SERCA, where it binds calcium (important for muscle function), might become less functional during IR. This malfunction might be caused by problems with how protons interact with SERCA.

This study also suggests a potential new target for treatment. By focusing on these calcium-binding sites in SERCA, researchers might be able to develop new drugs to prevent IR injury. These drugs could potentially reduce competition between protons and calcium for binding sites on SERCA, thus lowering oxidative stress (cellular damage) and high phosphate levels, which are both linked to IR injury.

**What are the potential implications of these results for your field of research?**

We showcased the capacity of involvement of metabolite sensitivity in the myofilament crossbridge cycling and SERCA pump modeling by simulating how SERCA malfunctions during IR, highlighting it as a potential new target for developing therapies to prevent heart damage. The model can consider various factors like changing levels of energetic molecules, oxygen and cellular pathways to evaluate potential therapies. Furthermore, the model sets a robust computational foundation for future works aiming at refining the electro-mechano-energetic coupling in cardiac cells.

**What are the main advantages and drawbacks of the experimental system you have used as it relates to the disease you are investigating?**

The main advantages of the methods of our work include: (1) introducing a novel oxygen dynamics formulation that links the extracellular oxygen concentration to intracellular ionic dynamics and energetics of the contraction; (2) offering a detailed biophysical framework that considers metabolite sensitivity and energetics of contraction; and (3) offering a novel model for the mechanism of action of LEVO and validating the model simulations in IR arrhythmic conditions.

The main drawback is that due to the very limited data availability on hiPSC-CM mitochondrial dynamics, our model also lacks this energetic component. Furthermore, our model, called hiMCES, does not include an ATP-sensitive Na^+^-K^+^ pump (I_NaK_) formulation; therefore, the reflection of I_NaK_ in the oxygen dynamics formulation is simplified.

**Figure:**
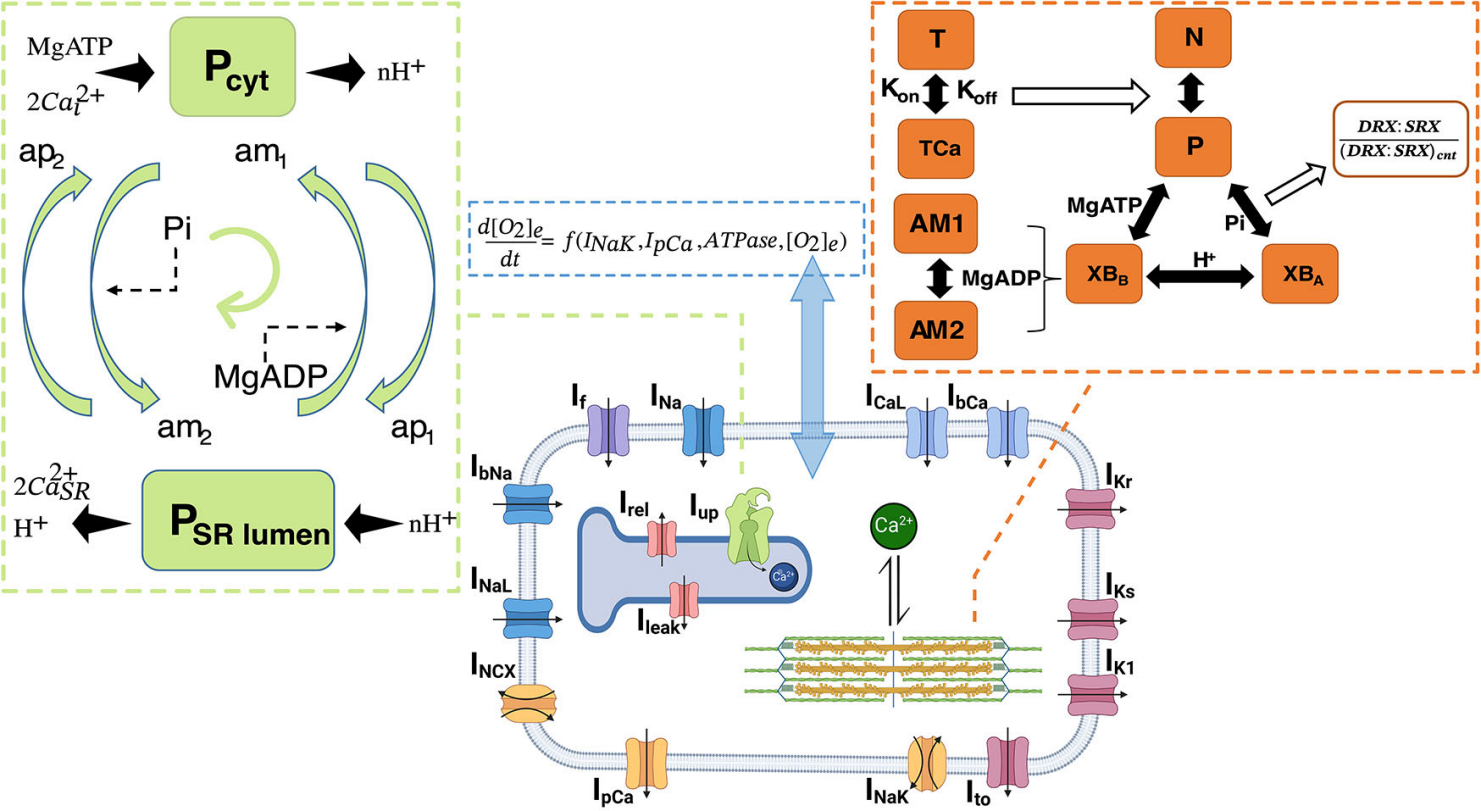
A schematic of the computational model of hiPSC cardiomyocytes addressing electro-mechano-energetic coupling.

**What has surprised you the most while conducting your research?**

The reparametrizing and optimization of different components of the mathematical model according to relevant experimental reports was the most challenging part. I was also surprised by the incredible capacity of detailed mathematical models in providing insight into subcellular crosstalks and the molecular mechanisms involved in IR and the drug-induced conditions in heart muscle cells.

**What do you think is the most significant challenge impacting your research at this time and how will this be addressed over the next 10 years?**

One of the most challenging features of these computational studies is the availability of experimental data using which we could validate our model parametrizations and simulation outcomes. Specifically, this becomes more problematic when the model addresses subcellular ionic interactions and key biomarkers at cellular level. I believe this issue will be properly addressed in computer modeling-guided chip designs for experimental protocols used for hiPSC-CM studies in the labs in the next 10 years and probably sooner than that.

**What changes do you think could improve the professional lives of scientists?**

The Faculty of Medicine and Health Technology of Tampere University provided a well-organized academic environment for doctoral students, which included expert follow-up groups and peer support. In my opinion, optimizing and refining mentoring programs for doctoral students and postdocs that provide candidate-specific guidance while boosting international collaborations and encouraging ambitious carrier goals would significantly benefit the professional lives of scientists.

**What's next for you?**

I would like to pursue a computational biophysical endeavor on the electro-mechano-energetic coupling in heart muscle cells. In particular, I am fascinated by the mathematical modeling of mitochondria, their role in various cardiac diseases such as heart failure and mutation-specific hypertrophic cardiomyopathy, and the potential for discovering new therapeutic targets.
